# Information Theoretic Characterization of Uncertainty Distinguishes Surprise From Accuracy Signals in the Brain

**DOI:** 10.3389/frai.2020.00005

**Published:** 2020-02-28

**Authors:** Leyla Loued-Khenissi, Kerstin Preuschoff

**Affiliations:** ^1^Brain Mind Institute, École Polytechnique Fédérale de Lausanne, Lausanne, Switzerland; ^2^Swiss Center for Affective Sciences, University of Geneva, Geneva, Switzerland; ^3^Geneva Finance Research Institute, University of Geneva, Geneva, Switzerland

**Keywords:** uncertainty, information theory, surprise, confidence, probabilistic brain, fMRI, decision-making

## Abstract

Uncertainty presents a problem for both human and machine decision-making. While utility maximization has traditionally been viewed as the motive force behind choice behavior, it has been theorized that uncertainty minimization may supersede reward motivation. Beyond reward, decisions are guided by belief, i.e., confidence-weighted expectations. Evidence challenging a belief evokes surprise, which signals a deviation from expectation (stimulus-bound surprise) but also provides an information gain. To support the theory that uncertainty minimization is an essential drive for the brain, we probe the neural trace of uncertainty-related decision variables, namely confidence, surprise, and information gain, in a discrete decision with a deterministic outcome. Confidence and surprise were elicited with a gambling task administered in a functional magnetic resonance imaging experiment, where agents start with a uniform probability distribution, transition to a non-uniform probabilistic state, and end in a fully certain state. After controlling for reward expectation, we find confidence, taken as the negative entropy of a trial, correlates with a response in the hippocampus and temporal lobe. Stimulus-bound surprise, taken as Shannon information, correlates with responses in the insula and striatum. In addition, we also find a neural response to a measure of information gain captured by a confidence error, a quantity we dub accuracy. BOLD responses to accuracy were found in the cerebellum and precuneus, after controlling for reward prediction errors and stimulus-bound surprise at the same time point. Our results suggest that, even absent an overt need for learning, the human brain expends energy on information gain and uncertainty minimization.

## 1. Introduction

Uncertainty is a feature of an agent's interaction with the environment that is both pervasive and unavoidable. Its ubiquity therefore demands a place in an agent's decision-making calculus. But uncertainty emerges in different forms during a decision, each of which can be uniquely susceptible to dysfunction. During an initial deliberation phase, for instance, agents form a belief on a decision's outcome, which is graded by confidence (Kepecs and Mainen, 2012). An outcome that challenges beliefs yields surprise (Hsia, 1991; Nour et al., 2018; Munnich and Ranney, 2019). Both confidence and surprise relate to uncertainty in the environment but their characterization remains a topic of debate (Itti and Baldi, 2009; Baldi and Itti, 2010; Munnich et al., 2019). Surprise may generate at least two quantities: one relating to an event's frequency (stimulus-bound surprise), and another back-propagating information gain that fine-tunes initial beliefs (model update) (Lorini and Castelfranchi, 2007; Itti and Baldi, 2009; Faraji et al., 2018). These two quantities together make up the uncertainty defined in the Free Energy Principle (Friston, 2010), whose minimization is theorized to be the brain's primary purpose (Schwartenbeck et al., 2015) and comprises a compelling theoretical framework for brain function. Questions on the neural characterization of different forms of uncertainty persist for both confidence (Pouget et al., 2016) and surprise (Munnich and Ranney, 2019). Current studies investigating uncertainty in the brain often rely on the notion of a Bayesian brain (Friston, 2012), where a probabilistic model of the world is built (the prior) and subsequently updated (posterior) through repeated interactions with the environment. In this paper, we seek to disentangle different aspects of uncertainty, namely confidence, as well as the dual facets of surprise, by applying a parsimonious, information theoretic model to BOLD response signals in a functional magnetic resonance imaging experiment. A neural response to these quantities would lend support for their emergence in the decision-making process.

### 1.1. Confidence

Human confidence is often thought of as a feeling but its mathematical definition has been extensively used in the fields of statistics and economics (Dominitz and Manski, 2004; Cesarini et al., 2006) and has more recently attracted interest in the neuroscience of decision-making (Kepecs et al., 2008; Kiani and Shadlen, 2009; Rolls et al., 2010; De Martino et al., 2013). Most studies on confidence in decision-making employ a subjective measure of post-decision confidence, obtained via self-report or inferred from reaction time (Kepecs and Mainen, 2012). Confidence arising prior to a decision outcome by contrast is a form of prediction uncertainty (Meyniel et al., 2015), or the second-order uncertainty coupled to a first-order expectation (Preuschoff et al., 2008a,b) and can be represented by the inverse variance (precision) (Yeung and Summerfield, 2014; Pouget et al., 2016) or the negative entropy of a probability distribution. Confidence is thought to weight both belief and the impact of its eventual violation: the more precise the prediction, the more significant its associated error (Feldman and Friston, 2010; Kwisthout et al., 2017). Neuroimaging studies on prediction uncertainty, specifically entropy and variance, have uncovered related BOLD responses in the hippocampus (Strange et al., 2005; Harrison et al., 2006; Davis et al., 2012), the striatum and insula (Preuschoff et al., 2006, 2008b; Mohr et al., 2010). Although confidence figures prominently in predictive processing theory (Friston et al., 2012; Barrett and Simmons, 2015), comparatively few neuroimaging studies have probed its unique contribution and neural representation. As confidence can confer an affective state (Sanders et al., 2016), it may correlate to anterior insular responses, and as it depends on prior knowledge, it may also relate to memory regions, such as the hippocampus and temporal lobe. Here, we seek a neural response to confidence as formalized by an information theoretic quantity, namely the negative entropy of a probability distribution, when an agent formulates an expectation.

### 1.2. Surprise

The error related to prediction uncertainty is commonly cast as surprise (Hayden et al., 2011; Preuschoff et al., 2011). The problem of surprise in both artificial intelligence and cognitive neuroscience hinges on its definition, which in turn opens a fraught discourse on its putative purpose (Munnich et al., 2019). From a phenomenological perspective, surprise is an organism's response to an unexpected change in her environment. Formal accounts of the phenomenon include Shannon surprise (Shannon, 1948); Bayesian surprise (Itti and Baldi, 2009); a predictive coding account of surprise [as absolute prediction error (Pearce and Hall, 1980) or risk prediction error (Preuschoff et al., 2011)]. These accounts share common features but are not perfectly correlated and, in some instances, can yield diverging values (Baldi and Itti, 2010). Broadly speaking, all but Bayesian Surprise can be considered “stimulus-bound” surprise, although both risk and absolute prediction error further integrate the value of an event, while Shannon Surprise is invariant to the latter. Itti and Baldi (2009) posit that an event can only be surprising if there is *post-hoc* evidence of learning; that is, the relevance of an event elicits surprise, not merely its improbability (Weaver; Faraji et al., 2018). Itti and Baldi formally distinguish Shannon surprise as stimulus-bound surprise and Bayesian surprise, an information gain represented by a Kullback-Leibler divergence (DKL) between prior and posterior beliefs (Itti and Baldi, 2009). They further argue that it is Bayesian Surprise that constitutes true surprise. However, one can argue that a rare event, formalized by Shannon surprise, is always relevant. The Free Energy framework (Friston, 2009) accounts for these distinct formulations of surprise by allowing for both stimulus-bound surprise and model update to constitute a measure of uncertainty (Free Energy), whose minimization is theorized to drive an agent (Schwartenbeck et al., 2015). In the brain, surprise as expectation violation correlates with BOLD responses in the salience network, including the anterior cingulate cortex and anterior insula (Uddin, 2014; Gogolla, 2017). Here, we seek to replicate previous results found in relation to stimulus-bound surprise specifically by applying an information theoretic account to the BOLD response, as the latter does not integrate the value of an event as risk and absolute prediction error do.

### 1.3. Information Gain

An unexpected outcome presents an opportunity to learn but more fundamentally, a chance to acquire knowledge. An intelligent agent should therefore exploit unexpected events so as to gain information. Information gain is commonly taken to be the Kullback–Leibler divergence, or relative entropy, which conforms to the notion of a Bayesian brain (Knill and Pouget, 2004) and therefore, implicitly, an assumption that certitude is never encountered (Basieva et al., 2017). However, an argument can be made that, in some instances and at higher levels of brain hierarchy, humans rely on approximate solutions and therefore can experience certitude. When a model cannot be further updated, or, in Markovian terms, when an agent reaches a terminal state, information gained from an event can be characterized as the difference between the truth (outcome) and the degree of prior belief (confidence), or absolute entropy (Shannon, 1948). What bridges the gap between belief and knowledge is an information gain and can be cast as an accuracy term. While accuracy is commonly taken as the difference between observed and (average) expected outcomes, we take it to be the difference between observed and the upper limit of expected outcomes (confidence). Thus, information gain may arise even if the model space is confined to one decision and can be defined for cases in which predictions are perfect, or outcomes are certain, as the self-evidence of a prediction (Parr et al., 2018), or the confirmation of a belief. For instance, suppose an agent invests in a given company's stock, estimating both it's future stock price and a confidence interval on that estimate. The agent wakes several years later to find the stock price has shot up suddenly, exceeding her expectations. The difference between the estimated and true stock price prompts a reward prediction error; the rarity of the event prompts surprise; and the discrepancy between the agent's confidence and the true outcome, or how far off the mark the agent was, represents a form of accuracy, or information gain. As in confidence, Bayesian formalization of information gain has gained considerable traction in recent years, but it can be argued that purely information theoretic accounts can simplify uncertainty quantification (Thornton, 2017). It is possible that the brain expends no resources on information gain if there is no future model to update however, a case can also be made for the curious brain, an information-hungry organism that collects and hoards evidence for possible future use. Here, we explore the neural response to a non-Bayesian information gain, which notably can be used in one-shot decisions.

### 1.4. Empirical Evidence of Stimulus-bound Surprise and Model Update

The dual aspect of surprise as both an alarm signal and a quantity of information is theoretically compelling, but less convincing in a human context. Stimulus-bound surprise necessarily calls on an autonomic response (Preuschoff et al., 2011), while an information gain need not. Several empirical studies have sought neural evidence of surprise's dual role. An examination of surprise models in P300 ERP signals finds Shannon information best explained data rather than a KL divergence, or a model that discounted forgetting across study blocks (Mars et al., 2008). Stimulus-bound rather than Bayesian surprise provided a better fit to the P300 ERP, widely viewed as a neural “surprise” signal, however, evidence of distinct neural systems correlating to stimulus-bound surprise and Bayesian surprise were found using fMRI (O'Reilly et al., 2013; Schwartenbeck et al., 2015; Kobayashi and Hsu, 2017). These studies suggest that, in humans (1) stimulus-bound surprise comprises a relevant phenomenon and that (2) a surprise-related learning signal also implicates a neural response. What remains unknown is whether a neural response reflecting information gain, distinct from a signed prediction error and stimulus-bound surprise, can be identified in the case of a one-shot decision process with a deterministic outcome where the Kullback–Leibler divergences cannot be computed. Such a signal can serve as a stand-in for subjective measures of post-decision confidence, bypassing report-related error and would also lend credence to the principle of uncertainty minimization as a primary neural drive.

In the following study, we examine three main questions in the context of value-based decision-making under uncertainty. We seek the neural representation of distinct but related uncertainty variables, notably confidence, surprise and accuracy. Specifically we hypothesize that (1) stimulus-bound surprise will elicit a BOLD response in the insula, striatum, anterior cingulate as in previous studies pertaining to error detection; (2) that confidence signals will be reflected in the insula, striatum and hippocampus, as entropy and risk have in other studies; (3) that accuracy signals will incur a unique BOLD response after accounting for reward prediction error and stimulus-bound surprise at the same time point. We test our hypotheses using fMRI within the context of a gambling paradigm that elicits both uncertainty predictions as well as their concomitant errors while controlling for reward, motivational, learning and motor effects. Capturing these quantities in the brain can inform on the human decision-making process, and notably provide guidance in where the process can fail. Several clinical populations show signs of dysfunctional decision-making (Pellicano and Burr, 2012; Limongi et al., 2018), yet the precise nature of these lapses in judgment remains difficult to quantify. By the same token, a more detailed description of the human decision-making process can guide efforts in artificial intelligence by providing more variables with which a machine can learn.

## 2. Materials and Methods

To examine our question of interest, we re-analyzed data from an auditory gambling task performed during fMRI acquisition. In the previous study, we sought commonalities of uncertainty processing in perception and value-based decision making task (Loued-Khenissi et al., 2020).

### 2.1. Participants

Twenty-nine healthy participants (10 F, average age 25.13 years) completed the experiment. Participants were recruited via paper and online advertisements targeting the student populations of Ecole Polytechnique Fédérale de Lausanne and Université de Lausanne. Exclusion criteria included metal implants, previous psychiatric illness, and psychotropic drug use within the past year. Inclusion criteria included proficiency in English.

### 2.2. Behavioral Task

**Figure 1:**
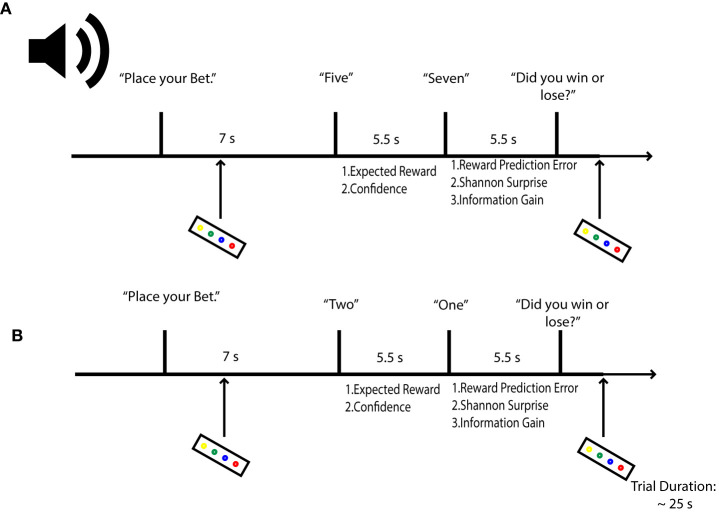
Probabilistic Gambling Task. Participants were asked to place bets on whether a second card draw from a deck of 10 would be higher or lower in value than a first card. Bets were placed before either card was revealed. Participants estimate their reward (expected value) and confidence (expected uncertainty) in the bet outcome after hearing card 1. After hearing card 2, agents can compute their reward prediction error; their stimulus-bound surprise and also their confidence error or information gain. Let us assume in the above example that a participant bets the second card will be lower. In **(A)**, confidence in the outcome will be low, given that the first card is a five; surprise is also expected to be low when card 2 is revealed, but information will be high, as the second card can take on several states for each outcome (1–4 for lower values, 6–10 for higher values) relative to the first card. In **(B)**, a participant should be confident that she will lose, as there is only one card out of a possible 9 that can deliver a win; therefore, when the second card yields the improbably one, surprise is expected to be high. Concomitantly, information gain is expected to be low, as confidence in the outcome had to be high.

To induce our target uncertainty variables, we employed an auditory version of a gambling task that has previously yielded responses to both prediction uncertainty and surprise (Preuschoff et al., 2006, 2008b). In the task, participants were asked to bet on the outcome of a card game. Starting with an initial endowment of 25 CHF ( 25 USD), participants bet 1 CHF that a second card drawn from a deck of 10 cards would be higher or lower than a first card. Bets were placed prior to any card being sounded. After the bet, the two cards were revealed sequentially, with a time lag of 5.5 s between their sounding. After the first card, participants could compute their chance of winning (predicted reward), as well as a confidence in their trial outcome prediction (predicted uncertainty). Once the second card was revealed, participants could assess their errors in reward and uncertainty prediction. Following the second card's sounding, participants were asked to report the bet's outcome, as a means of controlling for attention. Onsets for Cards 1 and 2 were separated by 5.5 s intervals, to better differentiate hemodynamic response function peaks relating to predictive and outcome phases of decision-making. A random jitter of 2–5 s was included following each trial. Each round of the card game lasted 25 s. To control for fatigue and attention, a penalty of 25 c was included for each missed bet and each missed or incorrect report. Participants viewed a black fixation cross on a gray screen during the imaging session, while stimuli were presented in pre-recorded wav files transmitted to MR compatible headphones, using Mac OS's text to speech function (Figure 1). The experimental task was written in Matlab (Matlab and Statistics Toolbox Release 2013a, TheMathWorks, Inc., Natick, Massachusetts, United States) using the Psychophysics toolbox (Kleiner, 2010). Participants were paid for their time at the end of the experimental session; task-related payout was reserved for a subsequent second experimental session, to lower rates of attrition.

### 2.3. Imaging Procedure

All neuroimaging data were acquired on a Siemens 3T Prisma at the Centre Hospitalier Universitaire Vaudois. Parameters for the EPI sequence were: 2D EPI, Multi-Echo sequence (3 echo times), 3 x 3 x 2.5 mm resolution, FOV = 192 mm; FA = 90 degrees, slice TR = 80 ms; TE = (17.4; 35.2; 53 ms); base resolution 64 mm; 34 slices; volume TR = 2.72 s; parallel acceleration mode = GRAPPA, with an acceleration factor = 2. At the end of the experimental session, anatomical T1 images were acquired with the following parameters: T1 MPRAGE, 1x1x1 mm resolution; FOV = 256 mm; slice TR/TE = 2 ms/2.39 ms; FA = 9 degrees; base resolution = 256 mm).

### 2.4. Image Preprocessing

Functional scans were preprocessed and analyzed using SPM12. Echo volumes were first summed to obtain one scan per TR. We then performed slice-timing correction and generated voxel displacement maps (VDM) to apply to functional volumes. Volumes were warped and realigned to the mean functional image using a 6 parameter (translations and rotations in space), rigid-body transformation to correct motion artifacts, before being bias-field corrected. Then individual T1 volumes were co-registered to the mean functional image using a rigid body model, estimated with mutual information. The T1 image was then segmented (6 class tissue probability maps) and normalized to MNI space using unified segmentation (Ashburner and Friston, 2005). These normalization parameters were then applied to functional volumes. Volumes were then smoothed with a Gaussian kernel of 8 mm FWHM.

### 2.5. Mathematical Models

**Figure 2:**
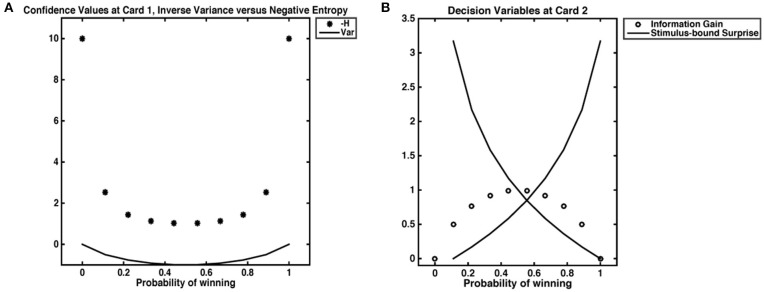
Decision variables. **(A)** Confidence as negative entropy or inverse variance. When outcomes are certain, neither inverse variance nor negative entropy are defined. However, approximating negative entropy by 0 yields a value that is ordinal to the next highest levels of confidence, while approximating 0 variance with an ϵ of 0.001 gives a value that does not scale with others confidence values. **(B)** We show the relationship between stimulus-bound surprise and information gain. Confidence is at its lowest when the probability of a win is 0.5; in such an instance, an agent has the most information to gain but does not experience the least (or most) surprise. Highest surprise is reserved for instances where confidence was high, such as cases where the probability of a win is 0.9; in such an instance, a loss would necessarily incur high Shannon surprise.

The task employed was designed to evoke probabilistic inferences in participants. The decision variables derived below are based on the probability distribution of winning (or losing) a gamble. Our computational model for reward prediction at card 1 reflects the average expected reward given the bet placed (higher or lower), and card 1's value (Preuschoff et al., 2011). The reward prediction error at card 2 reflects the trial outcome (win or loss) minus the reward prediction. Confidence is taken as the negative entropy H of outcome probability distributions after Card 1. This quantity is always negative and tends, when H = 0, toward 0. While negative entropy and inverse variance are often used interchangeably to quantify uncertainty and are numerically equivalent for most cases in our dataset, the inverse variance is necessarily undefined when ρ = 0. One could approximate such “infinite” confidence by setting ρ(0) = ϵ, however resultant values will 1) depend on ϵ; 2) yield a value for infinite confidence that is not ordinal to other values of confidence (Figure 2). At card 2, Shannon information quantifies stimulus-bound surprise, as the negative log of the probability of the observed outcome, *x*, given the bet placed, *b* and the value of Card 1, *c*. Finally, information gain was captured by the difference between the maximal value of confidence (certitude), minus confidence at Card 1. We take this maximal confidence to be 0; the information gain is thus always = <0, as it is the DKL; to differentiate this quantity from other forms of information gain, we call it accuracy. Because our task begins with an equal probability of outcome and ends with a terminal state that is independent of prior and future trial outcomes, we do not expect any learning to occur between trials. The trial begins with a flat prior and ends with a pseudo-deterministic outcome. Therefore, trials are assumed to be independent.

(1) $$\begin{array}{r} H=-p_{win}\cdot \log_{2}\left(p_{win}\right)-\left(1-p_{win}\right)\cdot \log_{2}\left(1-p_{win}\right) \end{array}$$

(2) $$\begin{array}{r} Confidence=-H \end{array}$$

(3) $$\begin{array}{r} Surprise=-\log_{2}\cdot \;\left(p\left(outcome|bet,card1\right)\right) \end{array}$$

(4) $$\begin{array}{r} Accuracy=0-Confidence \end{array}$$

### 2.6. Imaging Analysis

We performed a model-based analysis on our functional neuroimaging data. Specifically, we parametrically modulated onsets of interest by mathematical quantities described below. At the subject level, we constructed a general linear model including one regressor for sound activation (following onset of instructions to place the bet and to report the gamble outcome, modeled by a Dirac function); one regressor for motor response (including onsets for bet placement and outcome report, modeled as a Dirac function); a regressor for onsets of the first card's presentation (modeled as 5.5.s boxcar function), parametrically modulated first by reward prediction, followed by confidence; and a regressor for onsets of card 2's presentation (modeled as 5.5.s boxcar function), parametrically modulated first by the reward prediction error; second, by stimulus-bound surprise; and finally by an accuracy term. Parametric modulators were serially orthogonalized in the order described above, ensuring that related BOLD responses to specific decision-making variables reflect that variable's unique contribution to the signal. Also included in the model were 6 motion-related regressors of no interest. We note that BOLD responses to expected reward and reward prediction errors were not of primary interest to our study; they are nonetheless included in the general linear model so as to account for their unique contribution to the BOLD response, thereby allowing for the isolation of uncertainty-related variables. Onsets were convolved with the canonical hemodynamic response function. The time-series was high-pass filtered (128 s); autocorrelation was modeled by an AR(1) function. We performed *t*-tests at the single subject level on confidence, Shannon surprise and accuracy regressors. Individual contrast images were then pooled as estimates in a random-effects model. At the group level, we conducted non-parametric tests using the SnPM13 toolbox (10 000 permutations, variance smoothing = 8 mm).

## 3. Results

### 3.1. Behavioral Results

Twenty-five participants were included in the analysis. Behavioral data was not acquired for the first three participants. A fourth participant showed an error rate in excess of 30% (tallied from missed bets and reports, as well as incorrect reports) and was excluded from further analysis. Average task-related payout was 29.57 CHF; across all sessions and subjects, payoffs were in the range of 13-39 CHF. As the task designed included a truly random presentation of card pairs, we performed *post-hoc* analyses on potential differences for several variables of interest across sessions. We performed an *F*-test to determine if any one session contained more of one type of card value for card 1 and found no significant differences across sessions (*F* = 0, *p* = 0.996). We then performed tests on the mean differences of higher bets and lower bets across sessions and found no significant differences (*F* = 0.19, *p* = 0.8324 and *F* = 0.2, *p* = 0.8204, respectively), suggesting participants did not “switch” strategies across sessions. We also analyzed bet choices within blocks, by summing bet switches following a loss with bet persistence after a win, to assess the possible influence of prior bet outcomes. We find participants chose “non-strategic” bets more often (*t* = −3.01, *p* = 0.0035, df = 74), suggesting participants did not attempt to “learn” from previous outcomes. We also found a significant difference in bet choices with a higher likelihood for selecting a higher bet in all sessions (*F* = 34.69, *p* < 0.001).

### 3.2. Neuroimaging Results

We report results of voxels that remain significant when corrected for multiple comparisons, at a threshold of *p* = 0.05, FWE corrected at the whole brain level. Voxels were localized with the use of the Neuromorphometrics toolbox (Neuromorphometrics, Inc).

#### 3.2.1. Confidence at Card 1

**Figure 3:**
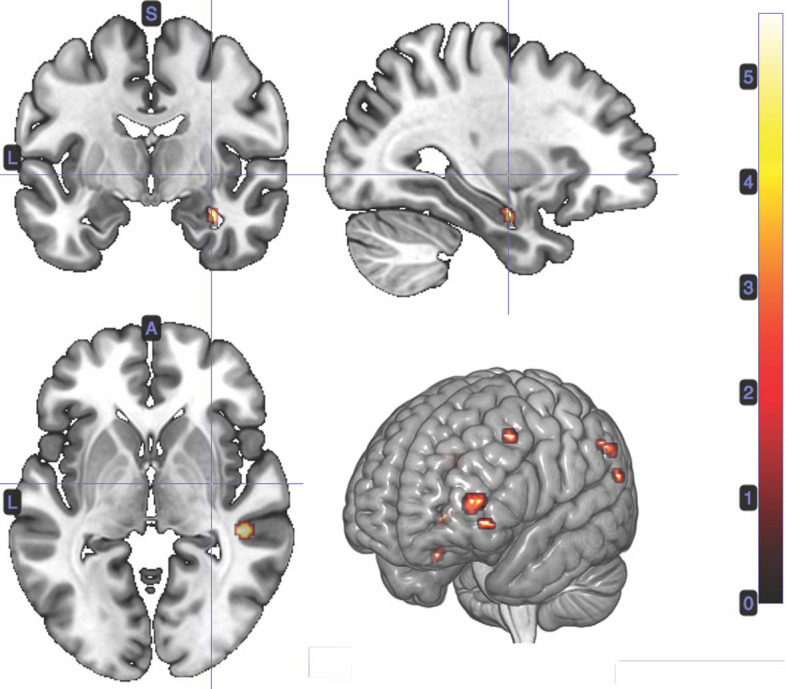
Statistical non-parametric map of significant clusters correlating to confidence in the interval between Card 1 and Card 2. Maps were thresholded with *p* = 0.05, FWE-corrected for multiple comparisons. The colorbar indicates *t*-values.

We performed a *t*-test on the onset of card 1's sounding for the prediction phase of the trial (duration = 5.5 s), parametrically modulated by confidence. Confidence here is orthogonal to reward prediction (experienced during the same time interval). We find a significant cluster in the right hippocampus; bilateral middle frontal gyrus; left supramarginal gyrus; right angular gyrus; right middle temporal gyrus; left superior temporal gyrus; and left inferior frontal gyrus. (Figure 3; Table 1).

**Table 1:** Statistics and locations of significant (*p* = 0.05, FWE-corrected) peaks and clusters related to confidence at Card 1.

**Confidence**						
***k***	**FWE**	***T***	***x***	***y***	***z***	**Region**
65	0.0022	5.61	−42	48	6	L MFG
32	0.0028	5.56	46	−32	−2	R MTG
32	0.006	5.33	−58	−50	38	L Supramarginal Gyrus
96	0.006	5.32	58	−54	28	R Angular Gyrus
23	0.0162	4.95	−44	18	44	L MFG
11	0.0188	4.9	32	−8	−22	R Hippocampus
12	0.0208	4.87	−48	44	−4	L IFG
9	0.0354	4.64	−60	−56	22	L Superior Temporal Gyrus
2	0.0368	4.63	−58	−42	40	L Supramarginal Gyrus
1	0.0464	4.54	62	−48	18	R Angular Gyrus

#### 3.2.2. Stimulus-Bound Surprise at Card 2

**Figure 4:**
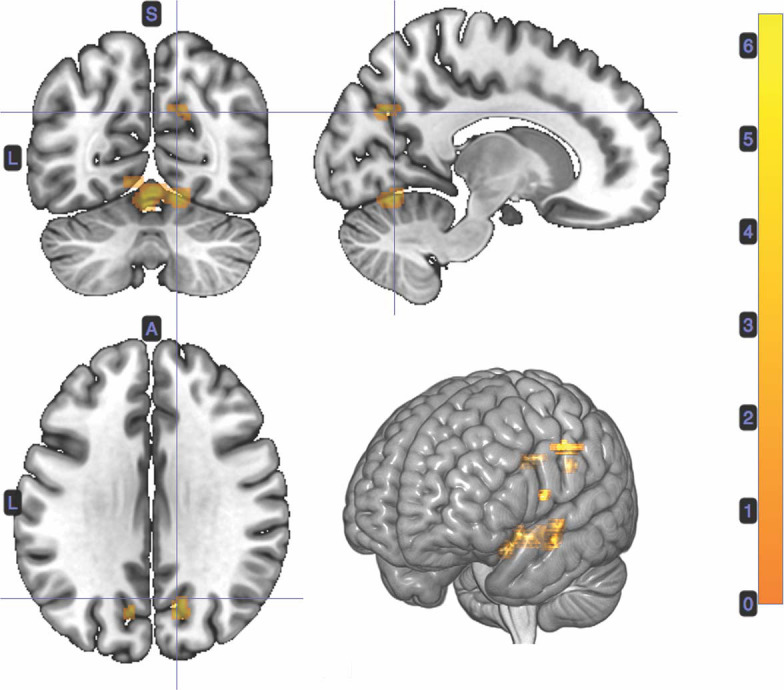
Statistical non-parametric map of significant clusters correlating to stimulus-surprise at trial outcome. Stimulus-bound surprise represented a second parametric modulator of Card 2's event onset, after accounting for the reward prediction error. Maps were thresholded with *p* = 0.05, FWE-corrected for multiple comparisons. The colorbar indicates *t*-values.

A *t*-test was performed on the onset of Card 2, parametrically modulated by stimulus-bound surprise of the trial for the duration between card 2's sounding and the outcome report (5.5 s). Significant clusters were found in expected regions, notably in the dorsal striatum (left putamen, right caudate); bilateral inferior frontal gyrii, extending into the anterior insula; left posterior cingulate cortex; bilateral medial temporal gyrii; and left supramarginal gyrus (Figure 4; Table 2).

**Table 2:** Statistics and locations of significant (*p* = 0.05, FWE-corrected) peaks and clusters related to stimulus-bound surprise at Card 2.

**Stimulus-bound Surprise**						
***k***	**FWE**	***T***	***x***	***y***	***z***	**Region**
655	0.0008	6.24	−22	−2	8	L Putamen
-	0.0012	5.85	−22	8	−6	–
-	0.002	5.57	−34	18	2	L Ains
738	0.0014	5.69	18	10	12	R Caudate
-	0.0022	5.51	44	20	−12	–
-	0.0022	5.49	24	−4	6	R Putamen
272	0.002	5.59	58	18	12	R IFG/Ains
-	0.0118	5.04	52	14	18	–
-	0.0126	5.02	52	30	16	–
69	0.0022	5.47	−62	−52	4	L MTG
38	0.007	5.19	0	−30	28	L PCG
108	0.0096	5.14	58	−56	6	R MTG
-	0.014	4.97	54	−46	12	–
110	0.0106	5.1	−50	38	6	L IFG
-	0.0156	4.91	−44	34	12	–
59	0.0128	5.01	-58	−52	24	L SupraMarginalGyrus
55	0.015	4.94	56	−36	−2	R MTG
57	0.016	4.9	−56	16	12	L IFG
-	0.021	4.82	−50	10	14	–

*Clusters with more than one significant peak in the same region are indicated with a dash*.

#### 3.2.3. Accuracy at Card 2

**Figure 5:**
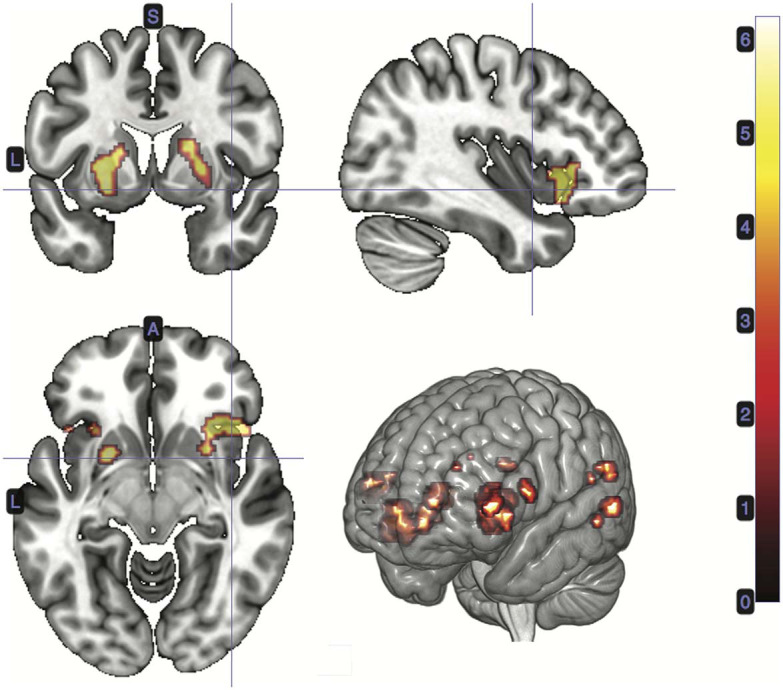
Statistical non-parametric map of significant clusters correlating to information gain at trial outcome. Information gain represented a third parametric modulator of Card 2's event onset, after accounting for the reward prediction error and stimulus-bound surprise. Maps were thresholded with *p* = 0.05, FWE-corrected for multiple comparisons. The colorbar indicates *t*-values.

A *t*-test was performed on the onset of Card 2, parametrically modulated by the accuracy of a trial, for a duration of 5.5 s. This quantity was included in the GLM as a third parametric modulator to Card 2's onset, following reward prediction error and stimulus-bound surprise. Significant voxels were found in the left supramarginal gyrus; bilateral; precuneus; bilateral cerebellum (exterior); and left central operculum (Figure 5; Table 3).

**Table 3:** Statistics and locations of significant (*p* = 0.05, FWE-corrected) peaks and clusters related to Information Gain (Accuracy) at Card 2.

**Information Gain (Accuracy)**						
***k***	**FWE**	***T***	***x***	***y***	***z***	**Region**
68		0.0002 6.34	−38	−38	38	L Supramarginal Gyrus
44	0.09	5.1	12	−70	32	R Precuneus
240	0.01	5.04	−10	-60	−10	L Cerebellum
-	0.0136	4.93	16	−64	−12	–
-	0.0158	4.87	24	−58	−20	–
17	0.0288	4.68	−12	−72	28	L Precuneus
6	0.0386	4.56	−42	−14	12	L Central Operculum/Posterior Insula
1	0.0486	4.46	−44	−28	40	L Post CentralGyrus

*Clusters with more than one significant peak in the same region are labeled with a dash*.

#### 3.2.4. Learning Across Trials

The experimental paradigm employed assumes no learning occurs across trials. Where there may be a learning effect is in the unlikely event that a subject counts card pairs as they are presented, because each possible card pair is only presented once. Should a subject deduce that each card pair is only presented once and also retain card pair values in memory as the experiment proceeds, we may expect the model space to expand to the experimental session. We nonetheless controlled for the possibility that a subject counted cards during the experimental sessions by designing a second GLM that differed from that described above only in swapping information with a Bayesian update measure. We computed this Bayesian update measure by employing a Dirichlet counting process, as per Strange et al. (2005), where wins were counted across a session, and included this measure of learning or divergence in a general linear model as a parametric regressor at Card 2. No significant voxels emerged, even when lowering the threshold to *p* = 0.05, uncorrected.

(5) $$\begin{array}{r} p_{win_{i}}=\frac{\sum_{1}^{i}Wins+1}{\sum_{1}^{i}Outcomes+1} \end{array}$$

## 4. Discussion

The results above show that (1) confidence, as negative entropy, correlates with the hippocampus, a region previously linked to uncertainty processing; (2) stimulus-bound surprise elicits activity in the insula and striatum, replicating previous studies; (3) accuracy, as a measure of information gain sampled at the same timepoint as stimulus-bound surprise, elicits a BOLD response in distinct regions, namely the cerebellum and precuneus. By using a formal account of all three measures while controlling for reward-related decision variables as well as task-related phenomena, such as overt learning and motor action, we link confidence, surprise and information gain to distinct neural correlates using information theoretic accounts. The emergence of a BOLD response for these three quantities underlines uncertainty's importance in human decision-making and lends empirical support to the principles of both uncertainty minimization and evidence maximization in brain function (Hohwy, 2012; Fiorillo, 2017; Pezzulo and Friston, 2019). Moreover, the localization of neural responses to surprise and information gain closely mirror a recent fMRI study investigating the similar questions but with the use of a Bayesian model (Kobayashi and Hsu, 2017).

### 4.1. Confidence

In our study, both the hippocampus and temporal gyrus correlate with confidence measures, in line with our hypothesis. Our results support the notion that confidence occupies a particular role in decision-making variables (Friston, 2018; Kiani and Shadlen, 2009; Insabato et al., 2010; Pouget et al., 2016). Confidence measures in human studies often suffer from being a self-reported, subjective measure assessed *post-hoc*. Here, we examine an objective form of confidence, captured by the negative entropy computed during a passive, predictive phase of an event's outcome. As prediction is theorized to arise from integrating an incoming stimulus into prior knowledge (Clark, 2013), memory regions should be implicated in this phase of decision-making. Previous studies have found a BOLD response in the hippocampus for related measures of prediction uncertainty such as variability (Rigoli et al., 2019) and entropy (Strange et al., 2005; Harrison et al., 2006) but here we explicitly find hippocampal responses for confidence, and not entropy or risk. Further, by using negative entropy rather than inverse variance, we divorce this quantity from the expected mean; that is, confidence is invariant to the value of the prediction. Our results further add to the current body of knowledge pertaining to brain correlates of confidence because we employ a whole-brain rather than ROI-based analysis. Other areas correlating with confidence include parietal regions, namely bilateral angular and supramarginal gyri. Angular gyri have previously been implicated in decision-making under uncertainty in humans (Symmonds et al., 2011; Studer et al., 2014). In monkeys, parietal neurons have previously been found to encode perceptual confidence using an evidence accumulation model (drift diffusion) in rhesus monkeys (Kiani and Shadlen, 2009). Finally, parietal lesions in humans have been found to leave recollection unaltered, but to specifically impair memory confidence (Simons et al., 2010). It is noteworthy that none of the studies above explicitly model confidence as negative entropy, but nonetheless yield similar neuroanatomical correlates. While the parietal lobe was not a primary focus of our hypothesis on the neural correlates of confidence, results from the extant literature validate our use of an information theoretic model of confidence.

### 4.2. Stimulus-Bound Surprise

We find evidence of stimulus-bound surprise in the (posterior) cingulate cortex and anterior insula, regions thought to signal error detection and conflict (Ullsperger et al., 2010); and the striatum, all regions previously implicated in studies on surprise (Preuschoff et al., 2011; Kobayashi and Hsu, 2017) but not found in other studies investigating both stimulus-bound surprise and information gain (O'Reilly et al., 2013; Schwartenbeck et al., 2015). Our results reaffirm the neural relevance of event improbability decoupled from the nature of the event (gain or loss) and by extension, the likely behavioral pertinence of such outcomes. Here, by controlling for the contributions of both the reward prediction error and information gain to the BOLD response at the outcome of a trial, we can confidently assert that our measure of surprise captures error-detection free of a hedonic component. Significant responses in the temporal lobe, a memory region, further add credence to the predictive processing framework. Stimulus-bound surprise can only occur when an event is compared to a prior expectation, a state of affairs that necessitates a memory component.

### 4.3. Model Update, Learning, and Accuracy

Evidence of learning can best reflect an information gain. However, no learning is expected to occur in our task, and this by design. All trials start with an equal probability of winning, so no strategizing can occur and outcomes do not depend on previous trials. We nonetheless captured signals related to a quantity of information gain by measuring maximal minus predicted confidence, or absolute entropy (Shannon, 1948). To distinguish this quantity from a model update (O'Reilly et al., 2013) we call this error term *accuracy*. Absent such a signal, we can hypothesize that no information has been gained, which suggests an agent was certain in the predictive phase of a decision. Accuracy was reflected in the cuneus and cerebellum. The cuneus has previously been implicated in learning rates (Payzan-LeNestour et al., 2013) and belief updating (Kobayashi and Hsu, 2017), in line with results in our study and has also been implicated in perceptual evidence accumulation (Ploran et al., 2011; FitzGerald et al., 2015), however this region also correlated with stimulus-bound surprise in another fMRI study (O'Reilly et al., 2013). The cerebellum on the other hand showed the strongest response to information gain. While a role for the cerebellum has been hypothesized in learning (Doya, 2000; Friston and Buzsáki, 2016) and inferential processes (Blackwood et al., 2004; Friston and Buzsáki, 2016), it is not commonly viewed as a decision-making hub. Of note is the lack of BOLD response in the cingulate cortex, which contrasts with results found by O'Reilly et al. in their study (2013). The absence of a BOLD response in the cingulate cortex, a region commonly linked to conflict (Botvinick, 2007) underlines the quality of information gain, in that it need not stem from incongruence but more fundamentally as an acquisition of knowledge, even while being a “prediction error.” Our results underline the inherent value information has (Friston et al., 2012), for the brain would not expend energy on a response otherwise. The brain may collect seemingly useless information, for a potential future. The implication of information collection is not trivial: it supports the notion that an agent may want to maximize her entropy (Schwartenbeck et al., 2013) and in so doing “seek” surprise (Clark, 2018), or a state of expecting the unexpected (Sun et al., 2011. Indeed, those individuals with stronger signals relating to information gain may be cast as more adventurous, or risk-seeking (Kruschwitz et al., 2012).

### 4.4. Hypothesized Disruptions of the Probabilistic Brain

Elucidation of uncertainty decision-variables can help identify specific components of dysfunctional decision-making and learning, particularly in patient populations (Parr et al., 2018). Isolating a neural response to confidence alone, for instance, may help shed light on aberrant decision-making. A compromised ability to compute confidence may lie at the heart of pathologies such as obsessive-compulsive disorder (OCD) (Hermans et al., 2008; Vaghi et al., 2017) and anxiety (Grupe and Nitschke, 2013; Carleton, 2016). Therefore, one could probe a patient's response to confidence in the hippocampus to determine if it deviates from a healthy range. Both repetitive actions and negative outlooks (expecting the worst) may increase confidence, and therefore minimize (unpleasant) surprise in OCD and anxiety patients (Hein et al., 2019), respectively; but increasing confidence would also erroneously minimize information gain (Kwisthout et al., 2017) and therefore accuracy. While these strategies are maladaptive, they are not irrational; framing them in the context of aberrant computations offers a way to identify the specific sub-process causing distress (Parr et al., 2018). Probabilistic computation may also be compromised in autism (Sinha et al., 2014; Van de Cruys et al., 2014); and schizophrenia (Silverstein et al., 2017). For instance, autistic individuals overestimate the volatility of an uncertain environment (Lawson et al., 2017). A disorder where stimulus-bound surprise is not computed may result in apathy and flattened affect, a common symptom in schizophrenic patients. On the other hand, an inflated stimulus-bound surprise could overwhelm an agent, which may be a feature of autism. Difficulty acquiring information specifically by discounting the accuracy term above could impede an agent's change in belief. Similarly, too large an information gain signal could indicate false belief formation (Schwartenbeck et al., 2015). Therefore, the neural processing of each of the quantities probed above may contribute to a specific dysfunction in behavior. Simulations of agents with specific deficits can be conducted to predict pathological symptoms of different psychiatric disorders.

### 4.5. Uncertainty in Man and Machines

The findings above also impact questions in artificial intelligence (Macedo and Cardoso, 2001; Lorini and Castelfranchi, 2006; Lorini and Piunti, 2007). If artificial intelligence is modeled after human behavior (Lake et al., 2017) then formalizing and finding evidence of the processes deployed in human intelligence offers a more precise template to reproduce. The utility in endowing a an intelligent agent with uncertainty and model update computation is clear. Less convincing is the need to encode all forms of uncertainty-related variables. Humans need stimulus-bound surprise, as it prompts a fight or flight response, presumably in the face of death: updating a model may well be irrelevant in such a case, or at the very least, secondary. In machines however, a model update may be necessary and sufficient, while stimulus-bound surprise may be surperfluous. Another consideration with respect to artificial modeling of surprise is the inclusion of its affective component. Hedonic components of surprise, such as positive and negative valence, can be accounted for in the sign of the reward prediction error. However, human surprise is also tinged with a range of other graded emotions: joy, disappointment, disgust, horror, anger, awe and fear (Braem et al., 2015). One could engage in a thought experiment to identify cases when an artificial agent may need to “feel” different hues of surprise-specific emotion. There may be no concrete purpose in endowing an artificial agent with the capacity to encode awe, for instance.

### 4.6. Conclusions

Our aim was to employ information theory to model and decompose uncertainty signals in the brain. Studies investigating the probabilistic brain have primarily exploited Bayesian models (Knill and Pouget, 2004; Friston, 2012) however as seen in the study above, such models may not easily accommodate certitude or one-shot decisions. While our work cannot identify causal relationships between external stimuli and recorded BOLD signals, we nonetheless find a relationship between the two. Significant brain responses that correlate to specific formal accounts suggest such calculations are being performed. In finding distinct responses to confidence, surprise and information gain, we highlight the importance of uncertainty integration to the brain. In identifying a neural correlate of information gain for a discrete decision in particular we: 1) offer an alternative to the Bayesian Surprise model of the latter; 2) show that the brain seeks to maximize evidence even when there is no obvious reason to do so. The implications of our results may help refine efforts to model intelligent agents and provide specific measures to identify and quantify decision-making deficits in clinical populations.

## Data Availability Statement

The datasets generated for this study will not be made publicly available (1) At the time of data collection, participants were not asked permission for dissemination of their data. (2) There is no known way to completely anonymize neuroimaging data, as images allow for a crude form of facial reconstruction.

## Ethics Statement

The studies involving human participants were reviewed and approved by Swissethics (EC Vaud). The patients/participants provided their written informed consent to participate in this study.

## Author Contributions

LL-K designed the study, acquired the data, performed the analysis, and wrote the paper. KP designed the study and wrote the paper.

### Conflict of Interest

The authors declare that the research was conducted in the absence of any commercial or financial relationships that could be construed as a potential conflict of interest.
